# Comparison of Double Lateral Osteotomy and Asymmetric Dorsal Hump Reduction in Correction of Crooked Nose

**DOI:** 10.29252/wjps.9.3.290

**Published:** 2020-09

**Authors:** Ali Goljanian Tabrizi, Matin Ghazizadeh, Behrouz Barati, Sara Nouri

**Affiliations:** Department of Otorhinolaryngology, Head and Neck Surgery, Taleghani Hospital, Shahid Beheshti University of Medical Sciences, Tehran, Iran

**Keywords:** Crooked nose, Rhinoplasty, Osteotomy, Nasal septum, Hump reduction

## Abstract

**BACKGROUND:**

Several methods have been introduced to correct crooked nose during rhinoplasty. This study aimed to compare the final shape of nasal pyramid as well as patients’ satisfaction of the outcomes in two different rhinoplasty techniques.

**METHODS:**

Participants in this study underwent rhinoplasty with two different techniques of double lateral osteotomy in comparison with asymmetric dorsal hump reduction using rasp. Ninety patients were allocated in two groups by a quadruple block randomization. Patients were compared for the correction of nasal deviation 6 and 12 months after surgery. Their self-rated satisfaction with rhinoplasty outcome was also assessed using a researcher-made questionnaire.

**RESULTS:**

Crooked nose correction was performed in 45 patients in each surgery group. Primarily, the mean of nasal deviation in two study groups were relatively similar (159.83±22.37 degree in C-shaped group vs. 11.79±4.98 degree in I-shaped group). The changes in degree of deviation after rhinoplasty were statistically significant in both intervention groups. However, based on the shape of nasal curvature, double lateral osteotomy was superior in long term follow up in I-shaped curvatures. Patients’ post-operative satisfaction with their nasal appearance was higher in the group of double lateral osteotomy and they were less interested in re-surgery.

**CONCLUSION:**

The two rhinoplasty techniques were not statistically different in terms of changes in nasal deviations correction after the surgery. However, long term changes in I-shaped curvatures were more desirable in group of double lateral osteotomy. Use of double lateral osteotomy was associated with better satisfactory aesthetic outcomes among study participants.

## INTRODUCTION

Rhinoplasty is commonly performed to improve the nasal obstruction and refine the nasal shape.^1^ One of the most complex procedures in rhinoplasty is the correction of the crooked nose. There are multiple techniques for correcting the curvatures of the dorsal septum. One of the main challenges among otorhinolaryngologists and aesthetic surgeons is to decide which technique results in better surgical outcomes after rhinoplasty.^2^ Osteotomy is usually the preferred approach to treat prominent humps, while mild humps can be treated just by a rasp.^3^

Double lateral osteotomy is usually executed in rhinoplasties in which the nose is too wide, or in cases that the maxillary processes are asymmetrically joined with the nasal bones in the two sides of the nose; or in nasal surgeries for traumatic injuries. This technique is usually used to correct lateral nasal deviations and to reshape the convex nasal pyramid.^4^^,^^5^ Asymmetric rasp is another technique for dorsal hump reduction and to equalize the height and to obtain a straight looking nasal dorsum. This method is typically used for management of mild asymmetry of lateral walls and correction of the convexity in crooked noses. However, use of rasp sometimes leads to the development of bone irregularities.^6^

In some cases, it is crucial for surgeons to choose the most suitable technique for dorsal hump reduction and it could be difficult to decide about how to obtain the best aesthetic surgical outcome. Therefore, this study was conducted to evaluate the effectiveness of two different techniques of rhinoplasty, including; double lateral osteotomy in comparison with asymmetric dorsal hump reduction.

## MATERIALS AND METHODS

In the present randomized clinical trial (RCT), patients with crooked nose who attended Taleghani Hospital affiliated to Shahid Beheshti University of Medical Sciences, Tehran, Iran, for rhinoplasty between 2017 to 2019 were included in the study. The study protocol was approved by the Ethics Committee of Shahid Beheshti University of Medical Sciences (Ethics code: IR.SBMU.MSP.REC.1398.427) and registered in the Iranian RCT website (IRCT20200123046232N1). Before enrollment of patients into the study, the researcher explained the study objectives to the eligible patients and asked them to read and sign the written informed consent for their participation in the study. 

The study sample size was calculated with 45 patients in each group, considering alpha error of 0.05 and study power of 80%. Any patient with S-shaped deformities, patients with facial irregularities, and patients whose nasal bending was due to cartilage structures were not included in the study. Patients with trauma to the nose that required medical or surgical interventions were excluded from the study. The enrolled patients (n=90) were randomly assigned to two groups of 45 patients each by a computer-generated random table of quadruple block numbers.

Patients’ demographics, including age and sex were recorded from the hospital’s medical records. Type of nasal curvature (I vs. C-shaped) and primary degree of deviation were measured using facial imaging. In I-shaped crooked nose, there was no curvature in the nasal dorsum and there was just a one-sided deviation in the nasal appearance, while in C-shaped crooked nose, a curvature could be seen in the middle of the nasal dorsum. Deviation angles of the I-shaped nose were measured by defining the angle between the vertical midline from the nasion to the middle point of the upper lip and the line from nasion to the most prominent point of the nasal tip. 

In C-type nose, deviation angles between the line from nasion to the most prominent curvature and the line from this point to nasal tip were measured. The length of the nasal bone of each side was also measured in these patients. Patients were randomly assigned to a conventional asymmetric dorsal hump reduction (group A) or a double lateral osteotomy (group D). Deviation angles were re-measured 6 and 12 months after the operation. All three rhinology photographs (before surgery, 6 and 12 months after surgery) for each patient were provided in the same imaging center in Taleghani hospital on the basis of the standard protocol to minimize the measurement bias. The ideal angular value for I-shaped noses was considered to be 0° and 180° for C-shaped noses.^7^

Patients were compared in two study groups in terms of nasal deviation correction and satisfactory aesthetic outcomes. The research was done as a double blind study, so that subjects and researcher did not know the allocation of the two groups. The data were described using frequency (percentage) for categorical variables and mean±standard deviation (SD) for numeric variables. The results of Kolmogorov Smirnov test confirmed the normal distribution of numeric variables. Chi Square and Fisher’s Exact tests, and analysis of variance (ANOVA) were used to compare the intended outcomes between the study groups. For the statistical analysis, the statistical software of SPSS for Windows (version 21.0, IBM Corp. 2012. Armonk, NY: IBM Corp.) was used. P values of <0.05 were considered statistically significant.

## RESULTS

Data of 90 patients were analyzed. Mean age of all patients was 26.41±5.76 years (minimum of 18 and maximum of 47 years), and 66.7% of participants were females. Type of nasal dorsal deviation in 47 (52.2%) patients was C-shaped and I-shaped in 43 (47.8%). Table 1 demonstrates the patients’ characteristics in two surgical groups. As indicated, there were no differences in the characteristics of the studied patients in two groups (*p*>0.05). Two groups of double lateral osteotomy and asymmetric dorsal hump reduction did not show statistically significant difference in the primary degree of nasal deviation in either of curvature types (*p*>0.05).

**Table 1:** Patients’ demographic and nasal characteristics before rhinoplasty

**Variable**			**Asymmetric dorsal hump reduction by rasp**	**Double lateral osteotomy**	**P value**
Gender		Female	26 (57.8%)	34 (75.6%)	0.074^†^
		Male	19 (42.2%)	11 (24.4%)	
Type of nasal curvature		C-shaped	22 (48.9%)	25 (55.6%)	0.527^†^
		I-shaped	23 (51.1%)	20 (44.4%)	
Age (Mean±SD*)			25.44±5.00	27.38±6.33	0.112^††^
Pre-surgical grade of nasal deviation (Mean±SD*)	C-shaped		162.95±6.99	157.08±29.99	0.243^††^
	I-shaped		12.91±4.84	10.50±4.95	0.538^††^

^†^Results of Chi Square test, ^†† ^Results of independent t-test, *SD: Standard deviation.

**Fig. 1:**
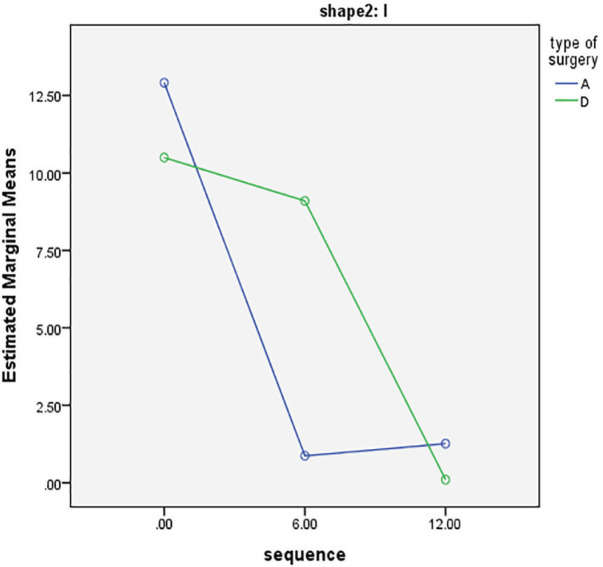
Comparison of changes in grade of nasal deviation 6 and 12 months in I-shaped deviations

Changes in degree of deviation 6 and 12 months after the rhinoplasty regardless of type of intervention were shown in Table 2 based on the shape of curvature. The grade of deviation significantly tended to zero in I-type category, while it increased toward 180 degree in the C-shaped group (*p*<0.001). Then, these measurements were compared in two groups of double lateral osteotomy and asymmetric dorsal hump reduction (Table 3). Results showed that in the I-shaped category, both interventions could significantly decrease the degree of deviation (*p*<0.05), but degree of deviation was slightly risen again after 12 months in group A (Figure 1). 

**Table 2:** Mean changes in grade of nasal deviation 6 and 12 months after surgery in all patients, based on the type of nasal curvature

**Type of nasal curvature**	**Grade of nasal deviation (Mean±SD)**			**P value**
	**Pre-surgery**	**6 months after surgery**	**12 months after surgery**	
I-shaped	11.79±3.23	4.69±3.23	0.72±3.23	<0.001^†^
C-shaped	159.82±3.09	175.53±3.09	175.10±3.09	<0.001^†^

^†^Results of ANOVA

**Table 3:** Comparison of mean changes in grade of nasal deviation 6 and 12 months in two surgical groups

**Type of nasal curvature**	**Type of surgery**	**Grade of nasal deviation (Mean±SD)**			**P value**
		**Pre-surgery**	**6 months after surgery**	**12 months after surgery**	
I-shaped	A	12.91±3.34	.86±3.34	1.26±3.34	0.023^†^
	D	10.5±3.59	9.10±3.59	0.1±3.59	
C-shaped	A	162.95±5.34	178.81±5.34	178±5.34	0.020^†^
	D	157.08±5.01	172.64±5.01	172.56±5.01	

^†^Results of ANOVA.

**Fig. 2:**
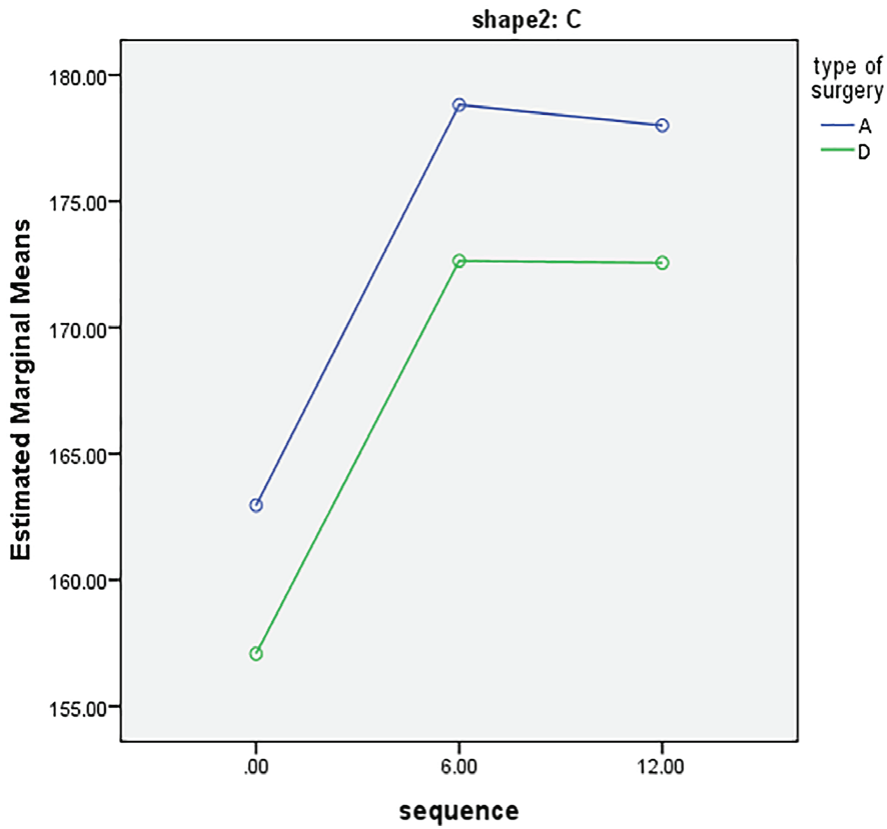
Comparison of changes in grade of nasal deviation 6 and 12 months in C-shaped deviations

**Fig. 3:**
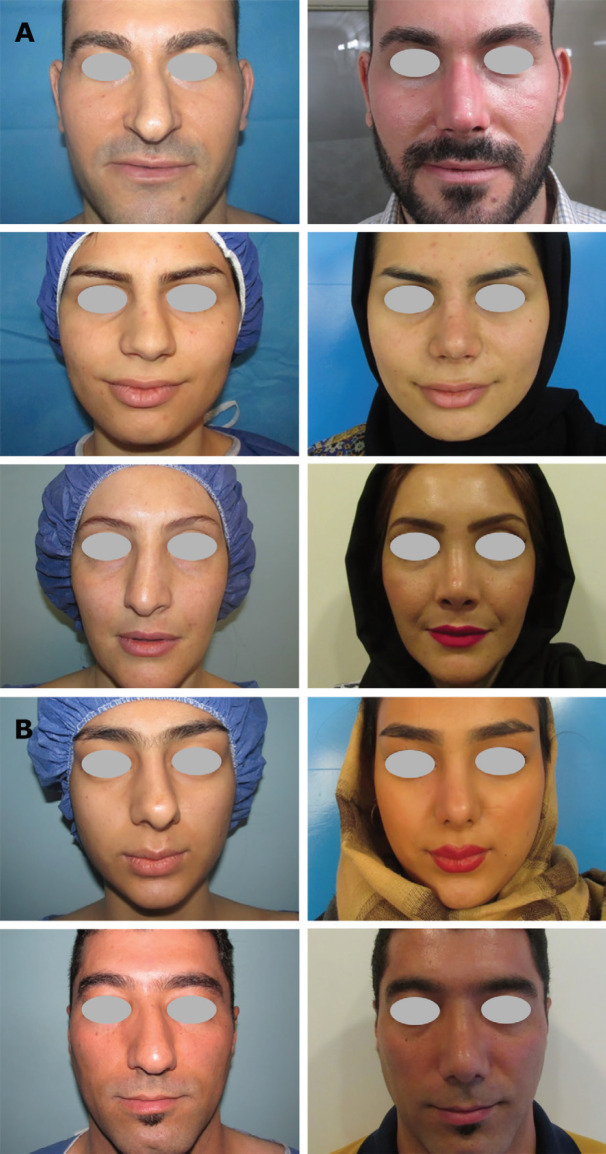
**A)** Comparison of nasal deviation pre and post-surgery in the group of double lateral osteotomy, and **B)** in the group of asymmetric dorsal hump reduction using rasp

**Fig. 4:**
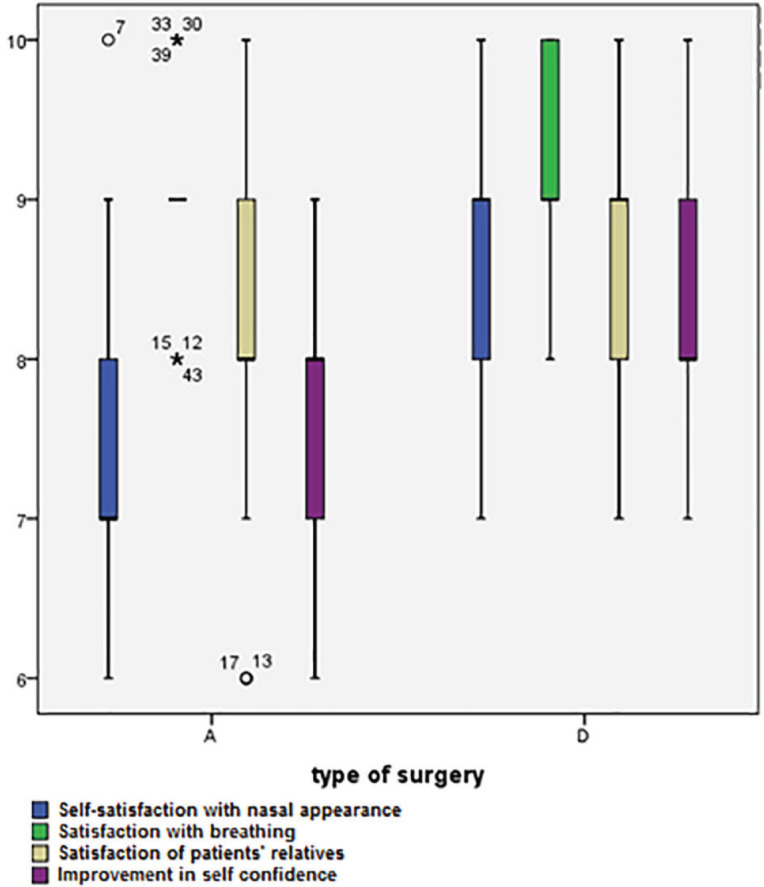
The postoperative satisfactory outcomes in two surgical groups

As well, Figure 2 shows that in C-shape group, the degree of nasal deviation was positively improved after surgery in both intervention groups (*p*=0.020). Figure 3-A demonstrates the surgery results in patients after 6 to 12 months in group D and Figures 3-B shows the surgery results after 12 months in group A. Patients’ satisfactions with the rhinoplasty outcomes were compared in Table 4. Results showed that patients who underwent double lateral osteotomy were more satisfied with surgery outcomes in all evaluated areas (*p*<0.05). The postoperative satisfactory outcomes of the group D compared with the group A in the boxplot (Figure 4). 

**Table 4:** Comparison of post-operative satisfaction in two surgical groups

**Post-operation satisfaction** **(Mean±SD)**		**Asymmetric dorsal hump reduction by rasp**	**Double lateral osteotomy**	**P value**
Self-satisfaction with nasal appearance		7.53±0.919	8.58±0.723	<0.001^†^
Satisfaction with breathing		9.07±0.580	9.07±0.751	1.000^†^
Satisfaction of patients’ relatives		8.13±0.968	8.56±0.893	0.034^†^
Improvement in self-confidence		7.53±0.694	8.20±0.726	<0.001^†^
Need for revision surgery	Yes	34 (75.6%)	2 (4.4%)	<0.001^††^
	No	11 (24.4%)	43 (95.6%)	

^†^Results of Independent T-test, ^††^Results of Fisher’s exact test.

## DISCUSSION

In the present study, we investigated the effect of application of double lateral osteotomy on correction of the crooked nose compared to asymmetric dorsal hump reduction using rasp and the results of the comparison of changes in degree of deviation showed the superiority of double lateral osteotomy, as it resulted in final lower degree of deviation after 12 months in I-shaped group. Although the mean changes after 6 and 12 months were statistically significant in both intervention groups, in the long term outcomes in group D were superior in I-shaped noses. 

In I-shaped crooked noses, asymmetric removal by rasp resulted in a significant reduction in degree of deviation after six months from 12.9° to 0.8°, but the results showed a recurrence in the following 12 months, while the improvement in group D remained steady. These results indicated that double lateral osteotomy was more effective than asymmetric removal by rasp in 12 months’ follow-up. Thereby, patients undergoing double lateral osteotomy mentioned less need for secondary rhinoplasty. 

Although we could not find any other study, comparing these two rhinoplasty techniques for correction of crooked noses, the results presented in our study is in line with that of a study conducted by Zucchini and colleagues, suggesting satisfactory score of Rhinoplasty Outcome Evaluations (ROE) after surgery, including nasal shape and final aesthetics outcomes were higher in osteotomy group.^3^

The reason could be due to the fact that the asymmetric reduction of dorsal hump by rasp may lead to the development of irregularities of the dorsum, whereas osteotomy led to a clearer cut and a more precise regular bone margins and finally better morphological correction of the nasal hump (this could be due to the effect of double lateral osteotomy on a more reliable relieving of tension vectors in the deviated nose). One other factor that could lead to osteotomy approach was the possibility of catastrophic aesthetic results if using a dull rasp that can cause much trauma to the dorsal skin.

Looking at the subjective outcomes of this study, in the group of double lateral osteotomy, patients’ attitude of rhinoplasty aesthetic outcomes and nasal breathing functions was more satisfactory than the patients who underwent asymmetric dorsal hump reduction. This result is nevertheless in contrast with a study that was suggesting post-operative scores of nasal appearance and its influence on patients’ self-confidence were higher in the rasp group. This study also showed that the rate of complications after surgery was higher among patients in osteotomy group.^3^

This result might be due to a greater traumatic effect of osteotomy on the pyramid dorsum. However, rate of complications from osteotomy surgeries was only 2%. Fortunately, satisfactory scores in all evaluated aspects were over medium in both genders who participated in this study. However, several studies have shown that females are more likely to precisely verbalize the morphologic or functional dissatisfaction.^8^^,^^9^ In our findings, the need for revision rhinoplasty was the most highlighted difference between double lateral osteotomy (4.4%) and asymmetric dorsal hump reduction (75.6%) that showed most patients in group D (95.6%) did not want to undergo revision. Several studies had discussed that most patients were usually unwilling to undergo a secondary operation for reasons such as fear of anesthesia or pain.^10^^-^^12^

Consequently, double lateral osteotomy could be a better selection in this regard. Despite the mentioned advantages of osteotomy approach for correction of the nasal deviation, rasping remains the best choice of curvature refinement in cases requiring minimal bony hump reduction. Besides, cases with moderate to large humps are mainly candidate for osteotomy techniques. The choice depends on the features of the bone and on the surgeon decision. However, it would be a great dilemma to select the best procedure in cases with mild but thick dorsal hump bone.^13^

The limitations of the present study included the one-year follow-up results of the patients, while longer follow-ups can indicate the long-term outcome results, such as the rate of reoperations. In addition, we did not record all possible objective outcomes of the surgery such as surgical procedure duration or other unpleasant aspects of rhinoplasty such as postoperative symptoms, bleeding, periorbital edema, pain, and ecchymosis.

## CONCLUSION

Double lateral osteotomy is a rhinoplasty technique that can be applied for correction of crooked nose. This method is not statistically different with asymmetric dorsal hump reduction in terms of changes in nasal deviations correction and both of these methods can significantly improve the grade of nasal deviation. However, findings showed that the use of double lateral osteotomy was superior in long term follow up in I-shaped curvatures. It was also associated with better satisfactory aesthetic outcomes among study participants and the patients who underwent osteotomy significantly reported less need for revision surgery.
